# A Portable Compact System for Laser Speckle Correlation Imaging of Artworks Using Projected Speckle Pattern

**DOI:** 10.3390/jimaging6110119

**Published:** 2020-11-06

**Authors:** Claudia Daffara, Elisa Marini

**Affiliations:** Department of Computer Science, University of Verona, Strada le Grazie 15, 37134 Verona, Italy; elisa.marini.4@studenti.unipd.it

**Keywords:** laser speckle imaging, speckle pattern, digital image correlation, nondestructive technique, artwork diagnostics, cultural heritage, portable system

## Abstract

Artworks have a layered structure subjected to alterations caused by various factors. The monitoring of defects at sub-millimeter scale may be performed by laser interferometric techniques. The aim of this work was to develop a compact system to perform laser speckle imaging in situ for effective mapping of subsurface defects in paintings. The device was designed to be versatile with the possibility of optimizing the performance by easy parameters adjustment. The system exploits a laser speckle pattern generated through an optical diffuser and projected onto the artworks and image correlation techniques for the analysis of the speckle intensity pattern. A protocol for the optimal measurement was suggested, based on calibration curves for tuning the mean speckle size in the acquired intensity pattern. The system was validated in the analysis of detachments in an ancient painting model using a short pulse thermal stimulus to induce a surface deformation field and standard decorrelation algorithms for speckle pattern matching. The device is equipped with a compact thermal camera for preventing any overheating effects during the phase of the stimulus. The developed system represents a valuable nondestructive tool for artwork diagnostics, allowing the monitoring of subsurface defects in paintings in out-of-laboratory environment.

## 1. Introduction

Artworks are subjected to structural alterations induced by various factors such as aging, microclimatic conditions and conservation treatments. In particular, ancient paintings present a complex layered structure that is susceptible to surface and subsurface decay, such as cracks, delaminations and detachments. The monitoring of such “defects” at small scale (sub-millimeter) is one of the objectives of nondestructive testing techniques applied to the conservation field [1,2].

Holographic interferometry [3] is a powerful technique that allows the detection of surface displacements with sub-micrometric accuracy. It is non-contact and non-invasive, highly sensitive and wide-field. Moreover, algorithms for processing image data are available, allowing fast and quantitative nondestructive analysis of structural modifications of the object in real-time [4]. The drawback of interferometry, generally speaking, is its sensitivity to external vibrations, which makes achieving the optimal measurement conditions without controlled laboratory settings difficult. This issue and the requirement of optics-skilled operators represent the major obstacle for a widespread use of such technique in the routine diagnostics of artworks. More flexible interferometry-based techniques are represented by the speckle-based methods [5], such as Electronic Speckle Pattern Interferometry (ESPI) and Speckle Pattern Photography (SPP) [6,7].

The effectiveness of holographic interferometry and speckle-based techniques for the analysis of artworks is well demonstrated, as reviewed in [8,9,10,11,12,13]. The applications include the structural evaluation of restoration processes (such as consolidation, cleaning or protective treatments), the detection of alterations induced by aging (such as cracks or subsurface defects) and the real-time monitoring of deformation due to microclimate variations [14,15,16,17]. As the nature of many artifacts makes their transport to a dedicated facility not possible, many research efforts are addressing the problem of in situ diagnostics with portable speckle-based techniques [14,18,19,20,21,22,23,24,25,26].

The speckle effect arises from the interaction of a coherent radiation with a random structure, as is the case of reflection from a rough diffusing surface or transmission across a diffusive medium [27], leading to an observed intensity pattern with a typical granular appearance, resulting from the multi-interference of the dephased waves at microscopic scale. In particular, any deformation of the surface micro-morphology turns into a modification of the corresponding anchored speckle pattern, down to displacements in the order of multiples of the radiation wavelength [6].

Subsurface defects in artworks, such as a lack of adhesion among the constitutive layers, can be detected by inducing an opportune thermal stress. In correspondence of the detachment, the heat dispersion rate slows down the return to equilibrium causing an irregular deformation field, observed at surface level.

Speckle metrology, in short, allows measuring the object deformations by acquiring and analyzing a sequence of speckle patterns. The ESPI technique is based on a two-beam configuration similar to holographic interferometry (as such, it is highly sensitive, up to sub-micron scale) and has found advantageous applications in the conservation field [8,13,15,28,29,30]. The SPP technique, conversely, is performed without the reference beam, by acquiring the speckle intensity pattern generated by the object beam alone. By correlation analyses of speckle images acquired from the object in different states, for example before and after a thermal *stimulus*, the deformation field can be obtained. SPP is especially sensitive to in-plane displacement components and local tilting [6], down to tenth micrometers. For a full characterization of the displacement field, speckle imaging and speckle shear interferometry can be integrated in a single device [19]. Despite the lower sensitivity with respect to ESPI, the SPP technique has the advantage of simpler setup, acquisition procedure and stability requirements. Speckle image decorrelation was demonstrated effective in detecting defects on wooden paintings, frescoes and mosaics, also in comparison with ESPI, as documented in early work [18,31,32].

While speckle diagnostics on interferometry basis with portable ESPI setup is being largely reported in the above-mentioned literature, it seems that less interest has been given to speckle imaging-based methods and SPP setup in the specific field of artwork diagnostics. Some recent works are concerned with laser speckle imaging for the dynamic analysis of material processing in restoration (drying and solvent actions [14,26]).

The aim of our work was the development of an effective, portable and compact system for performing speckle correlation imaging on artworks in situ. The system exploits an indirect speckle pattern projected onto the painting instead of the (more usual) speckle pattern generated by the surface. The paper is focused on the instrumentation and optimal performance setup and presents a validation of the measurement protocol on a layered painting sample to demonstrate that the developed system is well dimensioned and that it is effective in the analysis of subsurface defects.

## 2. Material and Methods

### 2.1. An Effective System for Speckle Pattern Correlation Imaging of Artworks

This paper presents an effective system for laser speckle imaging of artworks. The following key features have driven the design of the system:

- the hardware device should be compact and portable, with a versatile setup configuration, for performing measurements in out-of-lab environments;
- the overall measurement process should be completely noninvasive, with any effect potentially harmful to the artwork (namely, the heating) under control;
- the sensitivity performance should be versatile, tailorable to the specific diagnostics by easy parameters adjustment;
- a measurement protocol should be defined, and, ideally, the workflow should be as simple as possible also for operators with non-optics (interferometry) skills; and
- the hardware and the software should be commercially available and cost-effective, with the advantage of a system eventually at disposal of a wider conservation science community.

**Figure 1:**
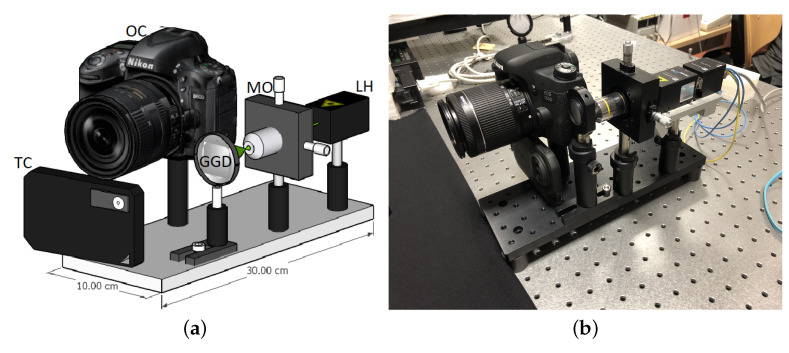
(**a**) Setup scheme. LH, laser head; MO, microscope objective; GGD, ground glass diffuser; OC, optical camera; TC, thermal camera. Approximate total weight: 3 kg. The supporting breadboard can be mounted on a stiff photographic tripod. (**b**) Picture of the device.

The designed system is shown in Figure 1. The setup is composed of the modules for generating, acquiring and processing the laser speckle pattern; the thermography module; and an external heating module. The modular design allows the fine adjustment of the components without affecting the alignment of non-involved parts. The laser source, thermal camera and photographic camera are fixed on a 10 × 30 cm optical breadboard and can be easily accessed and moved independently of one another. The system is compact and portable for in situ diagnostics: the total weight is about 3 kg and the optical breadboard can be easily mounted on a stiff photographic tripod. Of course, the setup with the acquisition camera positioned in free-standing, in a separate tripod, is possible.

**The laser speckle-generator module includes:**

- a DPSS laser source, 532 nm, 125 W power-controlled (RGB lasersystem);
- a microscope objective, serving as beam expander for a wide field of illumination (up to 1 m^2^) of the artwork; and
- an optical thin diffuser placed after the objective microscope and mounted on a stage to control the focused laser spot (as motivated later).

**The speckle image acquisition module includes:**

- a commercial high-resolution photographic camera able to image the speckle pattern (in this work we tested two CMOS cameras: Nikon D810 (7360 × 4912 pixel) coupled to a 50 mm lens with aperture *f*/9–11, controlled by the opensource software digiCamControl (Version 2.1.2) and Canon EOS 760D (6000 × 4000 pixel) coupled to a 18–55 mm zoom lens with aperture *f*/3.5–22, controlled by the smartphone app Camera Connect); and
- optical filters, with, optionally, a narrow-band filter for matching the laser wavelength and a linear polarizer for cleaning the speckle pattern.

**The speckle image correlation module includes:**

- software based on digital image correlation (DIC) for speckle pattern matching (in this work, we tested two standard methods, discussed below: a speckle decorrelation algorithm, used in cultural heritage applications [18], and a particle image velocimetry software, MatPIV (Version 1.7) [33], available as Matlab toolbox (Version R2014b))

**The thermography module includes:**

- a bolometer-based thermal camera (FLIR C2, 80 × 60 sensor in the infrared range 7.5 μm to 14 μm with thermal sensitivity <0.10 ${}^{\circ }C$), mounted next to the camera, for monitoring the effects of the thermal stimulus; and
- software (ResearchIR (Version 4.40.7.26)) for acquisition of radiometric thermal sequence in real-time.

**The external heating module includes:**

- a set of 750 W quartz tungsten infrared elements to apply a controlled step-heating pulse to the artwork, if the thermal stimulus is necessary; and
- a relay and a Theremino module to interface the lamps to the computer. It is important to shutter the lamps at the end of the stimulus, to avoid residual heating from the switch-off transient effect.

**Figure 2:**
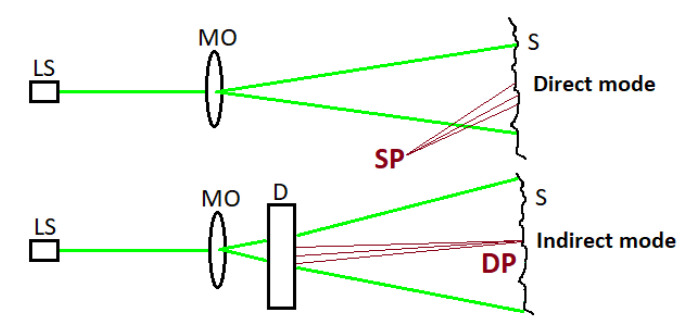
Schematic setup in direct and indirect configuration. LS, laser source; MO, microscopic objective; D, diffuser; S, surface; DP, diffuser speckle pattern; SP, surface speckle pattern.

The system can be configured in two working modes: the indirect mode, in which the speckle pattern is generated by the diffuser in transmission geometry and projected on the artwork, and the direct mode, without the diffuser, in which the speckle pattern is generated in reflection geometry by the artwork surface (Figure 2).

### 2.2. Setup Characterization Tests

Tailoring a speckle-based imaging application to the heritage field requires a thorough control of the setup as well as fine parameters tuning. The first laboratory tests were carried out to verify the suitability of the components in the main speckle-generator module: the laser source (effective output wavelength and wavelength variations with output power, beam divergence and polarization); the beam expander (suitability of microscopic objectives, with respect to transmission and expansion); the diffuser element (quality of the pattern, mainly with respect to transmission and diffusion level and intensity distribution, of different optical diffusers, from fine to coarse scattering).

The laser module was verified to produce a coherent, polarized and monochromatic beam. In the indirect configuration, a 10× microscopic objective (Olympus Plan Achromat (RMS10X)) coupled to the diffuser was able to provide a highly expanded beam (field of illumination of ∼1 m^2^ at 1 m distance), while higher magnification was needed in the direct configuration without the diffuser. Among the optical diffusers (Thorlabs N-BK7 Ground Glass; 120, 220, 600 and 1500 grit polishes), the finer grain was chosen for reducing the contribution from the zero-order beam, i.e., the component not diffracted away from the optical axis, preserving most of its energy and not properly diffused.

To address the problem of vibrations, the mirror-up mode of the camera must be enabled to avoid the blurring caused by the movement of the reflex mirror. Furthermore, the camera electronic front-curtain shutter could be employed instead of the mechanical one. In this regard, some tests were conducted, showing significantly reduced noise thanks to this simple expedient.

### 2.3. Motivation for the Thermography Module

Prolonged exposure to direct laser radiation during the measuring process could be harmful to delicate artifacts, in as far as it causes uncontrolled temperature rising. The thermal effect is mainly due to the directionality of the collimated laser beam and is reduced by the presence of the ground glass diffuser. Anyway, when applying the speckle technique to the analysis of subsurface defects, an external thermal solicitation is necessary for stressing the sample and inducing the displacement field. Following the request of the conservation scientists, to prevent any overheating effects in non-homogeneous artwork materials, we planned to acquire the artwork surface with a thermal camera, in continuous way, for the whole measuring session, thus allowing the temperature gradients to be monitored in real time. Beside monitoring the overheating, the thermal camera allows controlling the quality of the thermal stimulus during the excitation phase, i.e., that the infrared sources provide uniform and full-field irradiation and that the switch-off transient effect is effectively blocked (shuttering). The thermal load is quantified through a measurement of the raise of the surface temperature. Speckle methods were demonstrated on paintings using a weak thermal solicitation, with ΔT not exceeding few degrees [10,29,31,32].

The FLIR C2 mounted in the system is a low-cost compact thermal camera, recently available in the market, with access to the radiometric data; the small-size of the sensor is not a limiting factor to our application as this camera also acquires a visible image superimposed to the thermal one.

### 2.4. Performance Analysis

Since the laser source is characterized by high spatial and temporal coherence, the pattern originated from (direct configuration) or projected onto (indirect configuration) the surface is very stable in time and thus provides a valid “fingerprint” of the object. On the one hand, the speckle pattern is generated from the surface asperities at microscopic scale; as such, it is intrinsic to the surface itself. On the other hand, the speckle pattern is generated by the diffuser, which is a stationary random medium, and then projected onto the object, where, again, multi-interference at wavelength scale occurs and a “speckled-speckle” [27] pattern is formed. In principle, both the direct and the indirect configuration can be used for probing the structure of the surface and its deformation in time at micrometric scale. However, the pattern generated by the surface in the direct configuration (without the diffuser) is expected to have finer granularity than the projected one, thus be more difficult to handle.

Coming to the application of artworks analysis in situ, some considerations are needed concerning the specific focus of the diagnostics, the most important one, if the investigation is aimed at detecting surface defects or subsurface defects. Even if a rigid classification is not possible when dealing with artworks, by surface defects, we mean those related to a degradation of the surface texture, e.g., abrasion and painting *craquelure*, while, by subsurface defects, we mean those related to material inhomogeneities in the deeper structure, e.g., detachments in the painting or in the plaster layer, voids and fillings. Mapping surface defects thus necessarily requires the speckle pattern to carry intrinsic information from the surface. The presence of subsurface defects, instead, may be detected in extrinsic way from the relative displacement field of the surface as response to a stimulus. In this case, both the direct surface speckle pattern and the indirect projected speckle pattern can be effectively used. The point is that, strictly speaking, when the projected speckle pattern of the diffuser is used, there is, always, also a secondary speckle pattern produced by the surface.

The main two aspects which we are interested in are concerned with the morphology of the pattern, namely the extent of the granularity, and with the contrast of the intensity pattern.

#### 2.4.1. Sensitivity (Single Speckle Size)

The lower limit to the magnitude of the displacement that can be measured with the proposed setup is primarily determined by the mean speckle size, which in turn depends on the optical (lens aperture) and geometrical (distances) parameters of the setup. Following Goodman [34], the mean speckle size describes the extent of the spatial correlation of the speckle pattern and can be estimated as width of the autocorrelation function of the intensity at the observation plane. The pattern originated by the diffuser and projected on the artwork can be treated as “objective” speckles in free space (far field), in which the theoretical speckle size is given by [34]

(1) $$s_{\mathrm{obj}}\approx \frac{\lambda z}{D}$$

where λ is the laser wavelength, *z* is the object-to-viewing plane distance and *D* is the diameter of the region of the diffracting object (diffuser) illuminated by the laser.

Concerning the secondary speckle pattern originated at the artwork surface plane, we have that the speckled-speckle field, after propagating in free-space, is imaged by the camera, therefore it turns into the so-called “subjective” speckle, whose final size is determined by the lens aperture [34]

(2) $$s_{\mathrm{subj}}\approx \frac{\lambda v}{a}$$

where *v* is now the lens-to-image plane distance and *a* is the aperture diameter of the lens of the imaging system.

If the system is used without the diffuser, in the direct configuration, a single pattern is formed from the rough surface of the artwork and then imaged by the camera, as subjective speckle, with the speckle size thus determined again by Equation (2).

**Figure 3:**
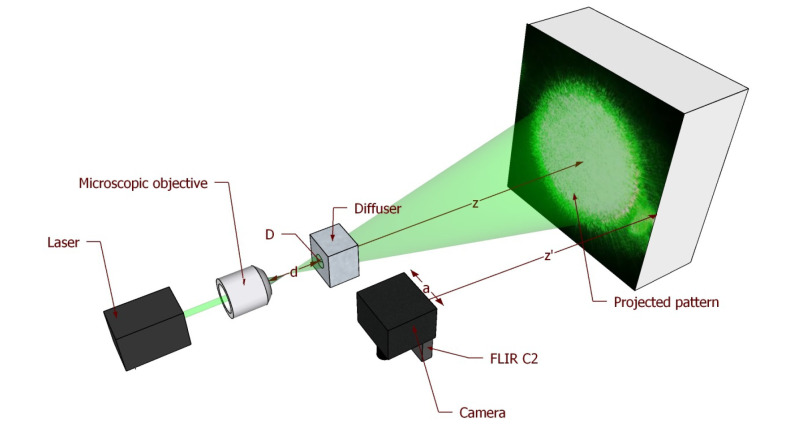
Setup scheme with the adjusting parameters: diffuser-to-object distance *z*; laser spot *D* (through the microscope working distance *d*); camera working distance $z^{\prime }$; and lens aperture *a*.

Generally speaking, for the aim of artworks’ diagnostics, the speckle size should vary in the range 100 μm to 700 μm to ensure a good sensitivity. The setup is designed to allow a fine tuning of the projected speckle size by adjusting the working distance of the microscope objective that focuses the laser spot on the diffuser, thus controlling the parameter *D* (see the setup scheme in Figure 3). The projected pattern is then reflected by the surface and imaged by the camera, so the diffuser speckles’ size changes according to the magnification factor of the camera lens.

It should be remarked that the above formulas for the mean speckle size are obtained under the common assumption that the speckle intensity displays negative exponential statistics [27]. The actual resolution of the whole apparatus depends on the entire optical chain (mainly, effects from camera detector integration and pixel sampling, partial depolarization of the scattered light, and non-uniform reflective surface). For this reason, having complete control on the size of the generated speckles, through an optimal parameters-tuning protocol, is fundamental for successive interpretation of the data. In this regard further studies are needed in order to get the full picture on how to originate patterns with specific, desired features by acting on the various parameters of the optical chain.

**Figure 4:**
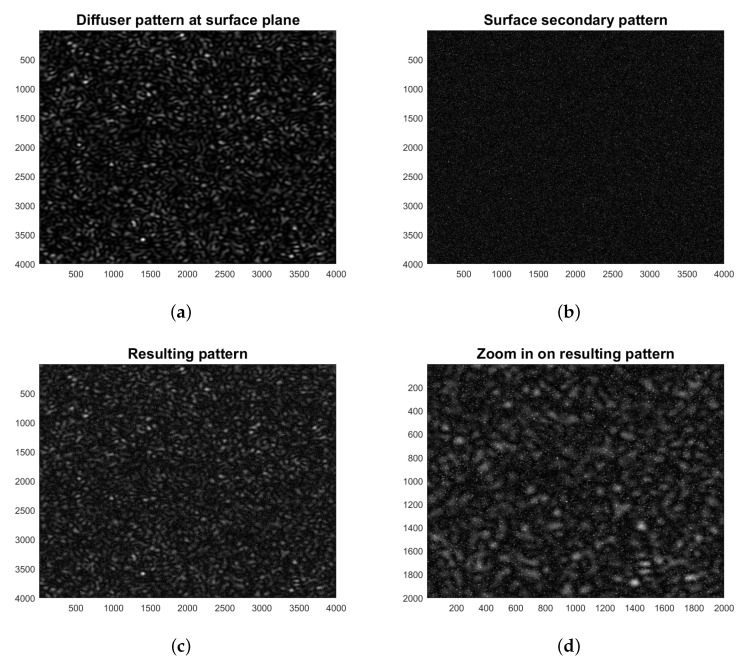
Speckle pattern simulations: (**a**) diffuser pattern at the surface plane; (**b**) surface secondary pattern; (**c**) superposition of the patterns as resulting after the reflection by the surface; and (**d**) zoom view.

In Figure 4, we show the appearance of the projected and secondary patterns and their superposition resulting at the camera observation plane. The simulations of the objective diffuser pattern and the subjective surface pattern were performed following the authors of [35,36].

#### 2.4.2. Optimization of Projected and Secondary Speckle Sizes

As explained above, the projected speckle pattern works as a kind of (random) structured light that is simply reflected by the artwork surface, which in turn generates its own speckle pattern. The resulting pattern observed through the camera of our setup appears as the superposition of two contributions: the indirect diffuser-generated pattern and the secondary surface-generated one (Figure 4c,d). Since the former is ultimately imaged after the reflection by the surface, the final ratio *r* between indirect speckles’ size (taking into account the magnification of the lens in Equation (1)) and secondary speckles’ size (Equation (2)) is independent of *v*:

(3) $$r\approx \frac{z}{z^{\prime }}\frac{a}{D}$$

In this work, we focus on the detection of subsurface defects by exploiting the information carried by the projected pattern. To this aim, we have to adjust the parameters of the setup so as to optimize the size of the diffuser speckles (the ones we are interested in) with respect to the surface ones. This way, we should minimize the information about the surface fine structure encoded in the acquired pattern while enhancing the signal related to the displacement field.

To aid the setting of the system, the behavior of the ratio *r* and its dependence on optical and distance parameters can be conveniently plotted and employed as indicative calibration curves. The following two practical situations are considered.

**Figure 5:**
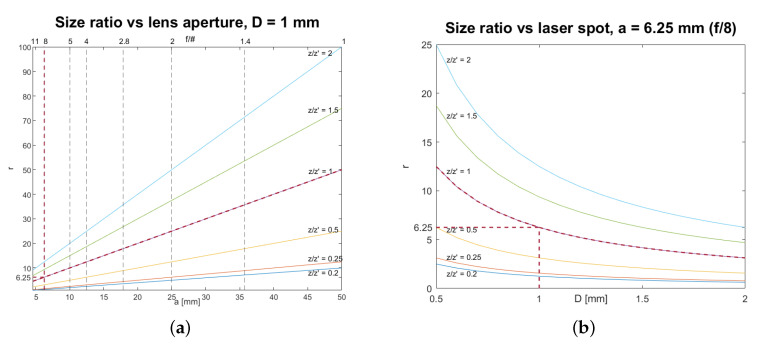
Calibration curves for the diffuser-over-surface speckles’ size ratio *r* for various values of $z/z^{\prime }$. (**a**) Fixing the laser spot *D* and moving the lens aperture *a*. On the bottom axis is the absolute lens aperture values and on the top axis is the f-stop values, *f*/#, which can be directly chosen from the camera, given for the case of the 50 mm lens. (**b**) Fixing the lens aperture *a* and moving the laser spot *D*. Red lines indicate the setup of the validation experiment ($z=z^{\prime }$) described in Section 3: for f= 50 mm, r=6.25 is achieved with f-number *f*/8.

1. In the first case, as depicted in Figure 5a, we observe that, once the size of the projected speckles (*D* and *z* in Equation (1)) has been chosen and an adequate pixel size has been set through $z^{\prime }$ to detect them, we only need to move *a* to regulate the ratio *r*, which varies linearly. A greater value of *D* would cause the family of curves to shift downward, while the opposite effect would arise from a decrease in the fixed value of *D*.
2. The second case, as depicted in Figure 5b, shows how we can change the ratio between the two patterns by adjusting *D* and without changing the pixel size (since $z^{\prime }$ is fixed, the camera field of view (FOV) does not change). This way, we can increase or decrease (in the limit of the resolution allowed by the fixed pixel size) the size of the projected speckles with respect to that of the secondary surface speckles, acting only on the diffuser speckles’ size. This is motivated by the fact that, once the field of illumination is chosen through the choice of *z* and the pixel size is determined through $z^{\prime }$, the value of the relative speckle size of the two patterns can be varied arbitrary through the choice of *D* and it decreases hyperbolically as *D* increases. Increasing (respectively decreasing) the value of *a* would shift the family of curves upward (respectively, downward).

### 2.5. Optimal Measurement Workflow

The above analysis suggests the following practical workflow for an optimal tuning of the setup parameters in the measurement session.

1. Identify the area to be inspected on the artwork surface. This will determine the field of illumination.
2. Set the diffuser-to-object distance *z* so as the diffuser projects the laser beam to uniformly cover the chosen area. According to Equation (1), the wider we make the field of illumination, by increasing *z*, the larger will result the projected speckles.
3. Set the laser spot *D* so that the diffuser-generated speckles have the desired size at the surface plane. This can be done by changing the distance *d* between objective and diffuser in the setup; *D* can be increased and tuned at will (up to diffuser diameter) to adjust the speckle size.
4. Set the camera-to-object distance $z^{\prime }$ to adjust the FOV and the pixel size. Decreasing $z^{\prime }$ will shrink the camera FOV increasing the resolution. In particular, the value of $z^{\prime }$ should allow to resolve details up to the order of the projected speckles’ size. More specifically, according to the Nyquist criterion, a pixel size should be such that there are at least two pixels per speckle, being the speckles’ size calculated on the basis of the needed diagnostic resolution.
5. The ratio of distances $z/z^{\prime }$ can be fixed as required by the out-of-lab condition, e.g., a museum environment, and the indirect to direct speckles’ size ratio *r* can be controlled through the laser spot *D*, or through the lens aperture *a*, following the calibration curves, respectively, in Figure 5a,b.
6. In the case of environment with limited or restricted spaces, for example in presence of scaffolding, the compact system configuration with camera and laser mounted in the same optical breadboard can be employed, corresponding to $z/z^{\prime }\approx 1$.

### 2.6. Speckle Image Correlation Analysis

As mentioned above, the data processing relies on two standard algorithms for pattern matching adapted to the SPP technique; the core of them is a local correlation principle. The first one, the speckle correlation (SC) algorithm [18], takes as input the intensity patterns acquired before and after the thermal stimulus and returns a correlation map where bright areas represent regions of high decorrelation, associated to anomalous in-plane displacements, while dark areas indicate regions where the pattern was almost unperturbed. The second one, MatPIV [33], is a particle image velocimetry tool, employed for the reconstruction of the surface average displacement field after the stimulus.

Briefly, the first algorithm works as follows: the speckle pattern acquired after the stimulus, $I_{\mathrm{mod}}\left(x,y\right)$, is subtracted from the one acquired before, $I_{\mathrm{ref}}\left(x,y\right)$, and then the difference is squared and averaged over an area containing many speckles, giving:

(4) $$Q\left(x,y\right)=\langle {\left[I_{\mathrm{ref}}\left(x,y\right)-I_{\mathrm{mod}}\left(x,y\right)\right]}^{2}\rangle$$

Under the assumptions of equal average intensities for the two patterns (due to stationarity), $\langle I_{ref}\left(x,y\right)\rangle =\langle I_{\mathrm{mod}}\left(x,y\right)\rangle =\langle I\left(x,y\right)\rangle$, and of negative exponential distribution for the acquired intensity patterns (fully developed speckle assumption),

(5) $$\rho_{c}\left(x,y\right)=\frac{Q(x,y)}{\langle I^{2}\left(x,y\right)\rangle }$$

is the complement-to-one of the (local) correlation coefficient ρ(x,y) of the intensity values measured at a point (x,y) of the two specklegrams, defined as the normalized (local) correlation function of the two patterns. The SC algorithm computes $\rho_{c}\left(x,y\right)$ and plots it as a correlation map for the two specklegrams.

In practice, the calculation of the complement-to-one of the correlation coefficient was performed through a discrete convolution of the image matrix with a small matrix (kernel), which performs an average in the neighborhoods of image points, as suggested in similar works [18].

As regards the MatPIV software, both the acquired patterns are divided into corresponding sub-regions $I_{\mathrm{ref}}^{i,j}$ and $I_{\mathrm{mod}}^{i,j}$ and, for each pair, the components (u,v) of their relative in-plane displacement are estimated by minimizing the cross-correlation between $I_{\mathrm{ref}}^{i,j}$ and $I_{\mathrm{mod}}^{i,j}$. After an average displacement is associated to each pair of corresponding sub-regions, the final result comes from the juxtaposition of such local displacement fields. Local deformations of the surface are thus mapped as local irregularities in the direction or in the intensity of the average displacement field, in the same regions where the correlation between the two images was dropping before.

## 3. Results

**Figure A1:**
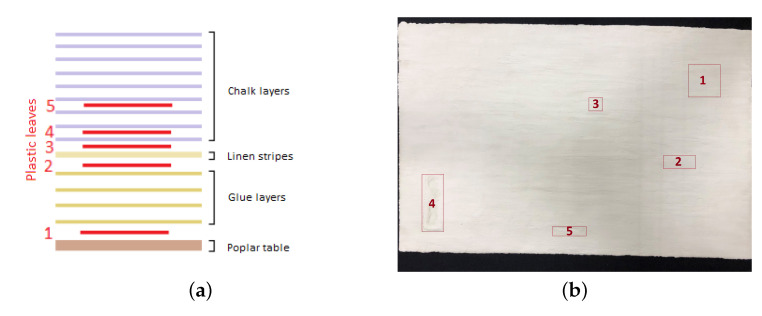
The fabricated painting model (40 × 60 cm): (**a**) scheme of the layered structure with the defects; and (**b**) picture with the position of the defective regions (numbered in red boxes).

The goal of this work was to propose an effective system for laser speckle pattern imaging (traditionally known as speckle photography) of artworks. Therefore, the developed prototype and the proposed optimal measurement protocol were validated in a typical context of artwork diagnostics by carrying out the experiment on a model of ancient painting with known hidden defects. The sample has the typical stratigraphy of Renaissance paintings and was prepared from the original receipts on materials and execution technique of *The Book of the Art* by Cennino Cennini [37], an Italian ancient treatise on paintings. A detailed description of the sample is given in Appendix A. The structural subsurface defects were modeled by inserting materials to break layer adhesion at various levels of the painting stratigraphy and in different positions, as shown in Figure A1.

**Figure 6:**
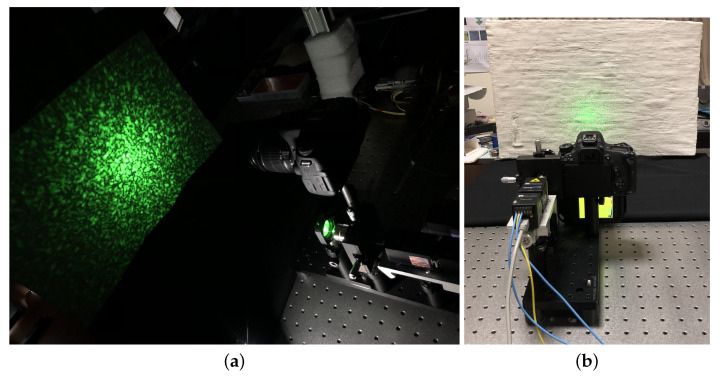
(**a**) Measurement setup with the system with camera, thermal camera and speckle module mounted in the same breadboard; and (**b**) back view with the lab lights on.

The system was used in the compact configuration, with the modules mounted in the same breadboard, and in indirect mode, i.e., projecting the speckle pattern on the painting surface. The two photographic cameras with fixed-focus and zoom lens, as described above, were tested. A working distance of 50 cm with a laser power of 60 mW was set to have a field of illumination of ≈40 cm diameter with a good quality projected speckle pattern, well matched to the FOV of the Canon camera at resolution of 40 μm (pixel size at object plane), assuring the optimal sampling of a 80 μm minimum detail (speckle size), suitable for the detection of a typical defective region in panel paintings. The results obtained with the Canon camera are comparable to those obtained with the Nikon camera, where the focal length was fixed at 50 mm and we adopted the same parameters, namely, an aperture value of f/8 and a working distance 50 cm, so as to have the same FOV with a higher resolution. A picture of the experiment setup is shown in Figure 6.

The thermal stimulus was applied to the painting using a single 750 W quartz tungsten infrared emitter. As discussed in Section 4.3, the duration of the thermal stress and the “response time” were critical parameters to optimize. Indeed, similar to any other active technique, i.e., based on the response of the system to an external solicitation (for example, infrared thermography), it is not possible to standardize such parameters in case of complex (and unknown) objects such as artworks. However, by quantifying the thermal load in the raise of the surface temperature, measured by the thermal camera, the duration of the stimulus can be set and initially suggested, for example, by previous works [10,29,31,38]. Therefore, different experiments were performed varying the duration of the heat stimulus (5 s, 15 s and 30s) up to a maximum ΔT∼5 ${}^{\circ }$C and acquiring the sequence of speckle patterns during the relaxation phase, after the lamp switch-off. In the case of the painting model, a short-pulse stimulus (5 s) was the most effective. This also satisfied the requirement of noninvasiveness.

In the design of DIC measurements, we followed the recommendations for the 2D-DIC given in “A Good Practices Guide for Digital Image Correlation” [39] by The International Digital Image Correlation Society. The object was positioned perpendicular to the optical axis; however, the surface of an ancient painting is not flat. The imaging system was used without any automatic adjusting, as auto-focus or apertures. As the employed DSLR cameras have an anti-alias filter, the images were not filtered. The intensity of the recorded speckle pattern was optimized by tuning the laser power, while keeping the gain of the camera low to minimize camera noise, as recommended.

**Figure 7:**
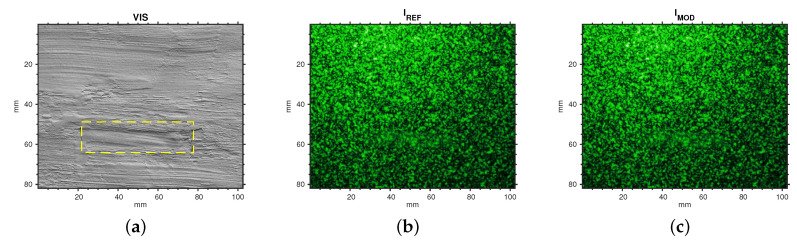
(**a**) ROI analyzed by the algorithms and defect position. Example of processed specklegrams: (**b**) before stimulus, used as reference pattern; and (**c**) after (5 s) pulse stimulus, modified pattern. Laser: 60 mW. Camera lens: 50 mm; exposure: 1/8 s; aperture: *f*/8; ISO: 100. Laser spot (*D*): 1 mm; diffuser-to-surface distance (*z*): 50 cm; camera-to-surface distance ($z^{\prime }$): 50 cm.

The analysis was performed on a region of the painting with a chosen defect (#5 in Figure A1). Figure 7 depicts the Region Of Interest (ROI) with a pair of speckle intensity pattern processed in the DIC analysis, the reference pattern $I_{\mathrm{ref}}$ (before the thermal stimulus) and the modified pattern $I_{\mathrm{mod}}$ (after the thermal stimulus). From the calibration curve (Figure 5), the lens setting provides an indicative ratio of the indirect to direct speckles of r=6.25.

### 3.1. Results with the SC Algorithm

**Figure 8:**
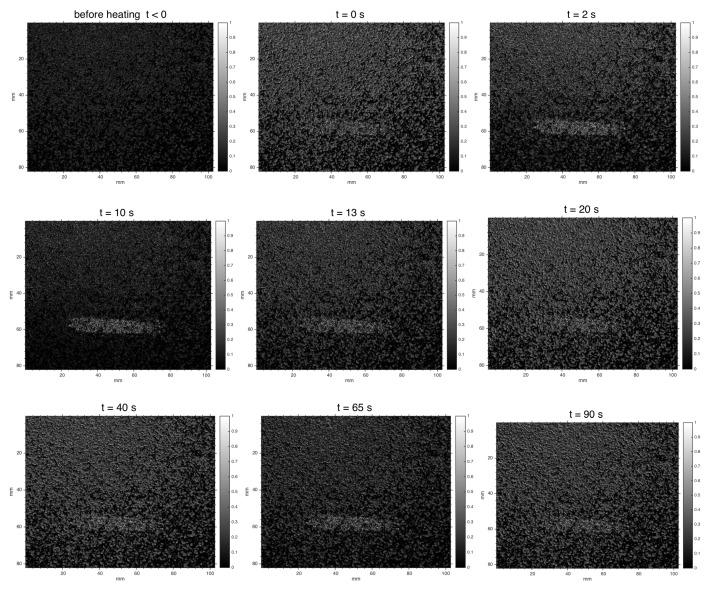
DIC results by SC algorithm. Laser speckle decorrelation map at different times: t=−10s, before thermal pulse stimulus (5 s); lamps switch-off at t=0; t>0, cooling phase.

Figure 8 reports the correlation map computed by the SC algorithm for some representative frames. There is also a video ( 100 s) availaible showing the behavior of the correlation map over time (Supplementary Material, Video S1: laser speckle decorrelation maps sequence). The sequence was obtained by processing, at significant step, the pairs of specklegram $I_{\mathrm{mod}}\left(t\right)$, $I_{\mathrm{ref}}$. Even if a continuous acquisition of the speckle activity is performed, one-beam speckle-intensity correlation methods require the off-line processing phase.

**Figure 9:**
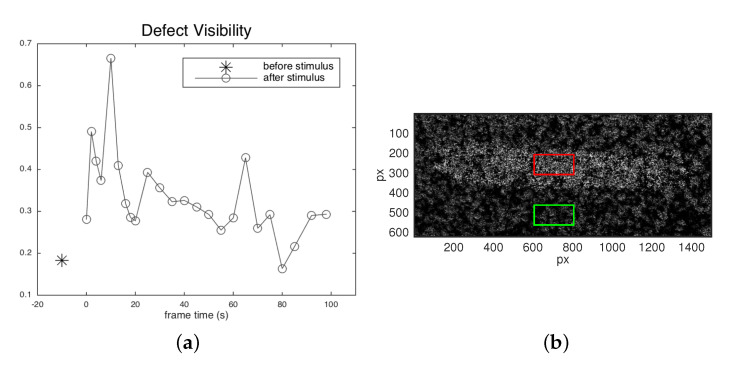
(**a**) Defect visibility at different frames, computed as Michelson contrast between the correlation coefficient averaged on a defective and on a sound ROI (Equation (6)). t=−10 s, before 5 s stimulus; t>0, relaxing phase after stimulus. (**b**) Zoom of the correlation map with the selected ROIs (100 × 200 points) on defective (red) and sound (green) region.

As one can see, a discontinuity in the gray level in the SC algorithm output of the normalized correlation coefficients (Equation (5)) well locates the defective region and its extension, where the painting surface undergoes an anomalous displacement. As expected, correlation computed on a pair of specklegrams of the sample in equilibrium before the thermal pulse does not reveal the defect. The bright decorrelated pixels that also appear in other regions, supposed to be non-defective, may be attributed to proper material inhomogeneities due to the handmade nature of the sample, as well as to external noise. As mentioned above, the effectiveness of speckle photography is based on the fact that the bulk defect, after thermal loading, determines a local irregular deformation of the surface and thus of the laser speckle activity. We see that in the case of pictorial detachments, which are positioned immediately beneath the surface, the defect is early detected at the beginning of the relaxing phase. The maximum visibility is observed at t=10 s, persisting across the 100 s sequence with a contrast that varies as the deformation of the defective region behaves different from the entire regular surface. In the correlation map computed by the SC algorithm, this can be quantified in the following visibility parameter

(6) $$\nu =\frac{{\langle \rho_{c}\rangle }_{\mathrm{def}}-{\langle \rho_{c}\rangle }_{\mathrm{ref}}}{{\langle \rho_{c}\rangle }_{\mathrm{def}}+{\langle \rho_{c}\rangle }_{\mathrm{ref}}}$$

that estimates the contrast in the correlation coefficient averaged in defective and reference (sound) region (Figure 9).

### 3.2. Results with the MatPIV Software

**Figure 10:**
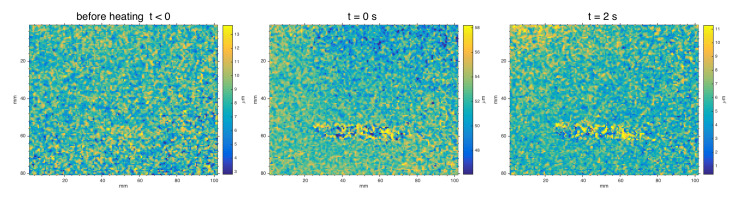

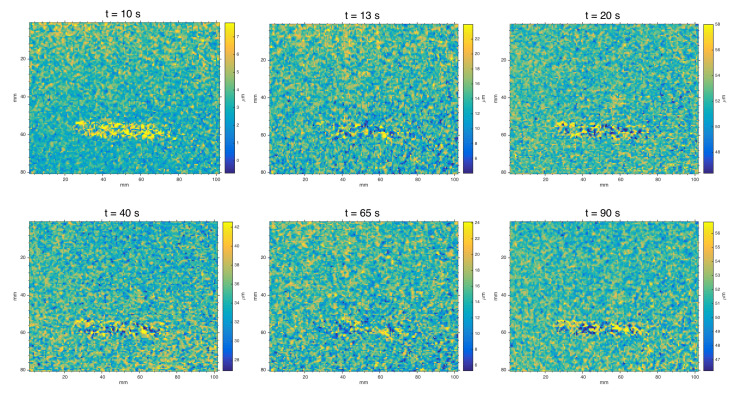
Absolute displacement map computed by MatPIV at different times: *t* = −10 s, before thermal pulse stimulus (5 s); lamps switch-off at *t* = 0; *t* > 0, cooling phase. The LUT is set to *μ* ± 3*σ* for best defect visualization. Noise-floor analysis in static images gives a mean *μ* ~ 8 μm and a variance error *σ* ~ 2 μm.

Figure 10 reports the displacement maps computed by the software MatPIV for some representative frames. There is also availaible a video ( 100 s) showing the behavior of the displacement field over time (Supplementary Material, Video S2: spatial displacement maps sequence)

**Figure 11:**
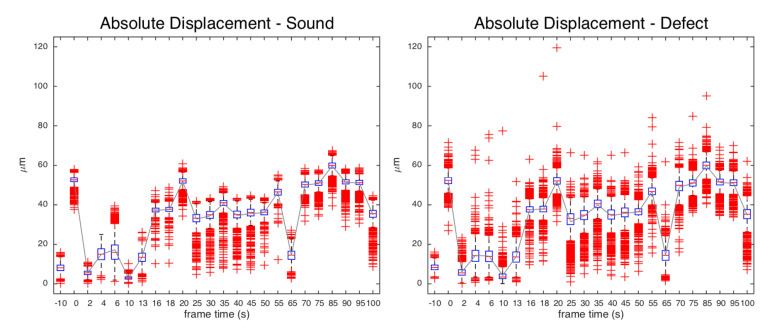
Boxplot to give an indication of the distribution of absolute displacements in defective and regular sound regions over time: t=−10 s, before 5 s stimulus; t>0, relaxing phase after stimulus.

**Figure 12:**
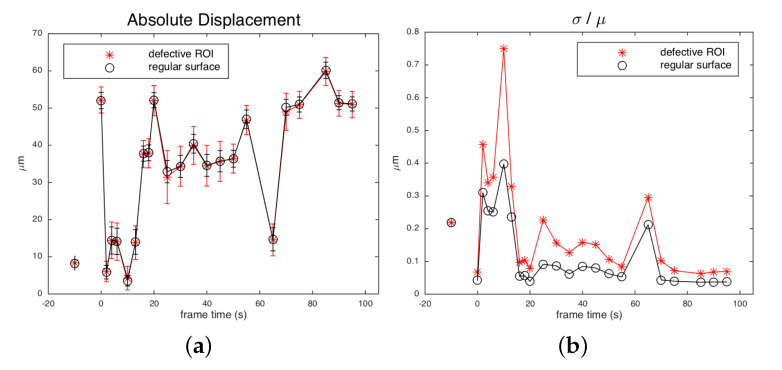
(**a**) Mean absolute displacement for defective and sound ROIs over time; and (**b**) coefficient of variation of the displacement distribution for the defective ROIs and for the regular surface over time.

MatPIV algorithm is successful too in detecting the defect, through the displacement map at sub-millimetric scale. The defect is early detected after the pulse stimulus as local anomalous behavior of the displacement field, and the visibility persists across the 100 s sequence. As expected, MatPIV computed on pair of specklegrams of the sample in equilibrium before the thermal pulse does not reveal the defect. Noise-floor analysis in static images (t<0) gives a mean μ∼8μm and a variance error σ∼2μm, mainly due to external vibration and camera noise. After the stimulus, bias errors are induced by heat waves, out-of-plane motion and vibration of the painting (large sample positioned in a vertical position). Anyway, it is interesting to examine the displacement distribution in the MatPIV maps. Figure 11 reports the boxplot of the absolute displacement over time, showing how the defective and reference sound regions exhibit the same mean displacement but a different dispersion in the relaxing phase. The coefficient of variation (relative standard deviation σ/μ) can be taken as indicator of the visibility of defect (Figure 12). The maximum visibility is observed at t=10s, similar to for the SC results, corresponding to small displacements but with a strong “decoupled” behavior of the local distribution in the defective region with respect to the regular surface.

### 3.3. Application of the Thermographic Module

**Figure 13:**
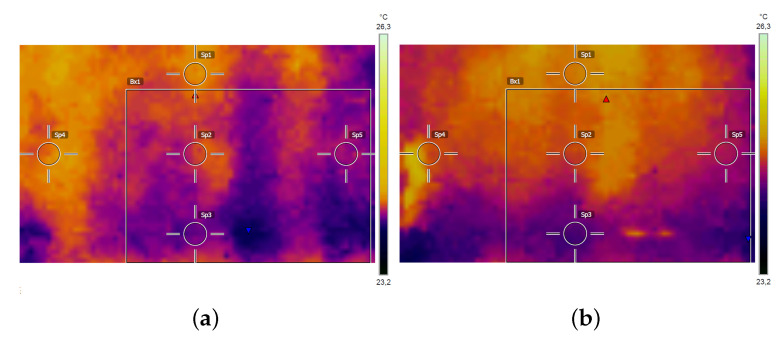
Thermal data: (**a**) (Sp1 = 24.2 ${}^{\circ }C$; Sp2 = 24.1 ${}^{\circ }C$; Sp3 = 23.9 ${}^{\circ }C$; Sp4 = 24.1 ${}^{\circ }C$; Sp5 = 24.0 ${}^{\circ }C$. Bx1: max = 24.2 ${}^{\circ }C$; min = 23.8 ${}^{\circ }C$; average = 24.0 ${}^{\circ }C$) before and (**b**) (Sp1 = 24.9 ${}^{\circ }C$; Sp2 = 24.7 ${}^{\circ }C$; Sp3 = 24.3 ${}^{\circ }C$; Sp4 = 24.7 ${}^{\circ }C$; Sp5 = 24.6 ${}^{\circ }C$. Bx1: max = 24.9 ${}^{\circ }C$; min = 24.1 ${}^{\circ }C$; average = 24.6 ${}^{\circ }C$) after the thermal stimulus of 5 s. The box indicates the investigated ROI.

With a thermal stimulus of 5 s and a lamp-to-surface distance of 40 cm, we observed a temperature increment of ∼1 ${}^{\circ }C$ right after the thermal solicitation (Figure 13). After 30 s waiting, the mean temperature was almost completely back to initial equilibrium. The mean temperature showed a maximum oscillation of 1 ${}^{\circ }C$ during the whole measurement process. When the laser alone was turned on, we measured an increase in temperature <1 ${}^{\circ }C$. Thermal emissivity was set to ε=0.90 for the involved material (gypsum). Being artworks surface characterized by inhomogeneous materials, roughness and decay degree, an assessment of the emissivity map for the calculation of the temperature field could be difficult. However, for many background materials, the tabulated values can be used.

## 4. Discussion

### 4.1. Discussion on Contrast Sensitivity

In the performance analysis, we focused on the mean speckle size of the speckle pattern. The other issue affecting the overall system sensitivity is the contrast sensitivity, which is primarily determined by the contrast of the speckle pattern, commonly defined as $C=\sigma_{I}/\mu_{I}$, the ratio of the standard deviation to the mean of the intensity distribution. It is well known [27] that a polarized and fully developed speckle field displays negative exponential intensity distribution with speckle contrast C=1.

The assumption of underlying negative exponential statistics for the acquired pattern is not always verified in practice, due to many factors such as the presence of the two scattering media (diffuser and object surface), the depolarization of the beam due to diffuse reflection at artwork surface, the integration of the camera sensor and the non-uniform reflectance typical of an artwork surface. It has been shown that [27]: the speckled-speckle pattern displays a conditional exponentially-driven intensity distribution with contrast $C=\sqrt{3}$; depolarization causes a degradation of the contrast due to the sum of independent (non-coherent) patterns; and the sum in intensity of patterns displays a Gamma density function, with contrast that decreases as the time of integration increases. Together with the speckle pattern morphology, the measured speckle contrast is thus affected by the actual statistics. Moreover, it is degraded by the camera acquisition process; anyway, it has been shown that, if care is taken, namely by sampling the speckle size above the Nyquist criterium, the contrast of the acquired speckle image approaches the theoretical limit and is maximized [40].

We addressed some of these issues with the aim of reducing their impact on the ideal negative exponential intensity distribution and to verify how the unavoidable deviation from the latter affects the overall performance of the technique. In doing this, the ratio between the diffuser speckles (expected to be fully developed) and the surface ones was set well over unity, so as to minimize the contribution of these latter to the acquired pattern. We placed a linear polarizing filter before the camera, after having verified that the diffuser does not depolarize the laser beam. The drawback, however, was the drop in intensity. As regards the integration process of the camera, numerical simulation tests have shown us that the statistics of the final pattern, and in particular its deviation from the exponential one, does not affect the performance of the software adopted for the DIC.

A study on the statistics of the intensity distribution and on the contrast of the speckle pattern in SPP application to artworks is planned as future development of this research.

### 4.2. Discussion on Non-Uniform Reflectance

**Figure 14:**
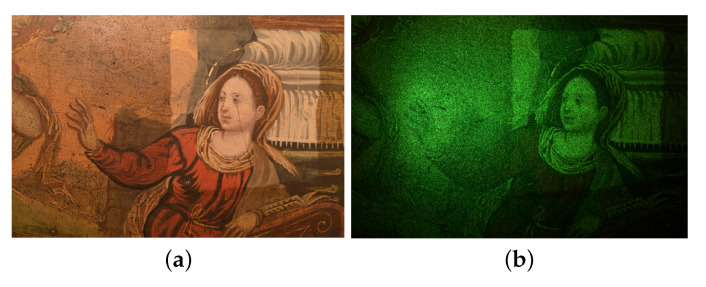
Non-uniform reflectance effect in a 16th century oil painting (anonymous, private collection): (**a**) visible image; and (**b**) speckle intensity pattern.

When dealing with artworks, a quite complex problem is the non-uniform reflectance of the polychromatic surface, as it affects the measured contrast locally. Figure 14 displays a speckle pattern projected on an ancient oil panel painting (anonymous, 16th century), showing the darker regions to absorb more incident radiation so that the speckle pattern is less or not detectable in their surrounding. Since the pattern is the unique information carrier, this could lead to a loss in information about surface modifications in the involved areas. To some extent, this issue can be addressed by controlling the external illumination and the power of the laser, but it remains a problem in the case of polychromatic and delicate artwork materials.

The study of the effect of non-uniform reflectance surface in SPP application to artworks is planned as future research direction.

### 4.3. Discussion on Thermal Stimulus and Response Time

The duration of the thermal pulse, and hence its intensity, is a critical parameter. In order for a sub-surface defect to manifest as speckle activity, the sample must undergo a physical stress strong enough to make evident, on the surface, the inhomogeneities due to the presence of bulk damages. The deeper is the defect, the more intense the solicitation must be. At the same time, a strict noninvasiveness requirement limits the thermal gradient on the surface to few degrees. The experiments pointed out that the general deformation to which the entire surface undergoes can be a detrimental factor, whenever it reaches the same order of magnitude as the defective area displacement. The ideal solicitation should maximize the deformation of the detachment, leaving at the same time the surrounding surface relatively unperturbed.

Since what is detected by SPP is a deformation of the surface, the response time is also a critical parameter. After the lamp switch-off, the energy stored in the surface must propagate to the inner defect. The resulting deformation intensity and time duration depend on the thermal capacity and expansion coefficient of the materials encountered in the stratigraphy. A detachment is a resistive defect that requires extra time to return to its equilibrium, and its deformed status, if recorded in this phase, emerges from the surrounding regions.

For the painting-like support, a $\Delta T∼1\;{}^{\circ }C$ (5 s pulse) was able to induce the visibility of sub-surface macroscopic detaches. Longer stimuli (15 s and 30s) did not improve the detectability; the most intense deformation field induced on the whole surface causes the local displacement of the defective area to be “hidden” by that of the regular surface. Unless the defect is very deep in the bulk, a short stimulus inducing a 2${}^{\circ }C$–3${}^{\circ }C$ gradient on the surface is suggested. Regarding the materials’ response, the experiments suggested an interval time in the order of 10 s for the superficial defects and in the order of 100 s for the deeper ones, in agreement with the literature. In the case of different support, i.e., from wooden to mural paintings, the conduction coefficients vary, and consequently the optimal acquisition times.

### 4.4. Discussion on Quantitative DIC

As mentioned above, the DIC measurements were designed following the good practices guide [39]. However, in speckle correlation applied to analysis of hidden sub-surface defects in artworks, some specific considerations must be made. The final objective is not the calculation of the derived field quantities, e.g., the displacement, but the localization of the defective area, which is revealed from an anomalous local behavior of the surface speckle activity with respect to the background. Moreover, the position of the sub-surface defect, as well as its nature, is mostly unknown; it is not possible to determine the expected surface deformation on a local ROI a priori, and the analysis should ideally be full-field. As a consequence, we have to face a trade-off between a large FOV and spatial resolution. The influence of optical system resolution on DIC uncertainties is discussed in [41]. DIC computation can be affected by many factors, related not only to the acquisition system and the laser speckle pattern but also to end-user decisions, such as the selection of the subset size to track the displacements [42]. One critical issue is that, for typical 2D-DIC applications, it is assumed that the sample remains planar at constant stand-off distance. Here, instead, the painting surface is subjected also to out-of-plane motion induced by the heat stimulus. In the case of artworks, it is not possible to estimate the out-of-plane deformation to compensate the in-plane measurements, as recommended by the good practice. Moreover, such out-of-plane motion, traced as fictitious in-plane absolute displacements, conveniently contributes to the irregular local behavior of the defective regions. Regarding the optics, the use of a bi-telecentric lens or of a long focal length is suggested to compensate the effect, allowing to solve the problem of magnification shifting and distortion.

## 5. Conclusions

A portable and very compact system for laser speckle imaging of artworks in situ is presented. The device was designed to be versatile, tailored to the needs of the art diagnostics field, with the possibility of optimizing the sensitivity performance by easy parameters adjustment. The system can operate in the indirect mode, in which the speckle pattern is generated through an optical diffuser and projected onto the artwork, and in the direct mode, in which the speckle pattern is generated by the artwork surface. The optimization of the optical setup through a tuneable speckle size (direct-surface and indirect-diffuser) was designed after a theoretical analysis of the performance based on statistical optics. A protocol for the optimal measurement was suggested, based on calibration curves for obtaining the desired mean speckle size in the acquired intensity pattern.

The system was validated in the analysis of subsurface defects in a model of ancient painting, using a short pulse thermal stimulus to induce a surface deformation field and the image correlation technique for the analysis of the sequence of speckle intensity patterns. To demonstrate that the developed system was well dimensioned and effective, the DIC was performed using two standard methods: the Speckle Correlation (SC) and the Particle Image Velocimetry (PIV) algorithms. The thermal loading induces irregular surface and sub-surface micro-motion, in-plane and out-of-plane, which is not traced quantitatively, but, if a proper (limited) thermal stress is used, a proper speckle size is used and a proper interrogation subset is used in DIC, the SPP technique is effective in differentiating the defective region as irregular local behavior with respect to the background. Even if a quantitative 2D-DIC was not the final objective (and not possible with complex multi-layered artworks), an analysis of the visibility of the defect was made in the correlation image sequence by SC and in the PIV displacements maps.

Following the requirement of noninvasiveness, a compact thermal camera was mounted on the system for monitoring the temperature of the artwork. The thermal camera allowed to face the critical issue of the optimization of the thermal stimulus: On the one hand, it allowed a quantification of the load intensity through a measurement of the surface temperature and an initial setting of the duration of the pulse (for example, following similar works in literature). On the other hand, it allowed the thermal solicitation to be maintained within the safe range of conservation standards. A surface temperature gradient ∼1 ${}^{\circ }$C induced by a short thermal pulse of 5 s was optimal for the detection of sub-surface detachments in the painting-like model, in accordance with the noninvasiveness requirement.

## Supplementary Materials

The following are available online at https://www.mdpi.com/2313-433X/6/11/119/s1, Video S1: laser speckle decorrelation maps sequence; Video S2: spatial displacement maps sequence.

## Abbreviations

The following abbreviations are used in this manuscript:

ESPI	Electronic Speckle Pattern Interferometry
SPP	Speckle Pattern Photography
DPSS	Diode-Pumped Solid-State
CMOS	Complementary Metal-Oxide Semiconductor
DIC	Digital Image Correlation
FOV	Field Of View
SC	Speckle Correlation
ROI	Region Of Interest

## Appendix A. The Painting Sample

The model of the Renaissance painting used in the experimental validation was fabricated following the traditional receipts on constitutive materials and execution technique contained in the ancient treatise *The Book of the Art* by Cennino Cennini [37]. The structural subsurface defects were obtained, as usual [10], by inserting materials with different thermal response.

We reproduced the process called *imprimitura*: a multi-layer poplar panel of 40 × 60 cm, preliminarily treated with four hands of solution of rabbit-skin glue with different dilution degrees, was covered with a number of rough linen stripes after they were immersed in the glue solution as well. After a drying time of two days, the table was smoothed with fine grain sandpaper, then some Bologna chalk powder was mixed to the rabbit-skin glue and the resulting mixture was warmed-up in a bain-marie and laid on the panel. After a drying time of three days, the surface of the table was smoothed again and eight layers of chalk and glue mixture were laid on it, each just before the complete drying of the previous one. After two final days of drying, the surface was smoothed one last time, obtaining the final sample, whose stratified structure is shown in Figure A1.

During the process, five thin plastic leaves were inserted at various levels of the stratigraphy and at different positions so as to simulate detachments of known dimensions, depth, and positions (Figure A1).
